# Model of Providing Assistive Technologies in Special Education Schools

**DOI:** 10.5539/gjhs.v8n1p36

**Published:** 2015-05-15

**Authors:** Suchitporn Lersilp, Supawadee Putthinoi, Nopasit Chakpitak

**Affiliations:** 1 Department of Occupational Therapy, Faculty of Associated Medical Sciences, Chiang Mai University, Chiang Mai, Thailand; 2 Chiang Mai University International College, Chiang Mai, Thailand

**Keywords:** model, assistive technology, students with disabilities, special education schools

## Abstract

Most students diagnosed with disabilities in Thai special education schools received assistive technologies, but this did not guarantee the greatest benefits. The purpose of this study was to survey the provision, use and needs of assistive technologies, as well as the perspectives of key informants regarding a model of providing them in special education schools. The participants were selected by the purposive sampling method, and they comprised 120 students with visual, physical, hearing or intellectual disabilities from four special education schools in Chiang Mai, Thailand; and 24 key informants such as parents or caregivers, teachers, school principals and school therapists. The instruments consisted of an assistive technology checklist and a semi-structured interview. Results showed that a category of assistive technologies was provided for students with disabilities, with the highest being “services”, followed by “media” and then “facilities”. Furthermore, mostly students with physical disabilities were provided with assistive technologies, but those with visual disabilities needed it more. Finally, the model of providing assistive technologies was composed of 5 components: Collaboration; Holistic perspective; Independent management of schools; Learning systems and a production manual for users; and Development of an assistive technology center, driven by 3 major sources such as Government and Private organizations, and Schools.

## 1. Introduction

### 1.1 Introduction to the Problem

“Students with disabilities” include children who have special needs in daily life activities, including self-care, education and social participation. For example, students with visual, hearing, physical, intellectual disabilities, etc., are different from those in general because of their physical and learning limitations. Despite the fact that these students have their limitations, they can achieve success when they have the opportunity to do so. In addition, human rights and equality are being promoted for people with disability issues, and due to this, opportunities for such students to study in the school system are increasing, instead of keeping them at home, as was the case in the past in Thailand. Providing assistive technologies can support students with disabilities, who face barriers in learning and participating with their peers in schools. In fact, assistive technologies are a way to enhance opportunities for this group of people (Massachusetts Department of Elementary and Secondary Education, 2012).

The Thai Ministerial Regulations for Provision of Assistive Technology, Media and Services in People with Disabilities regulates the meaning of “Assistive Technologies” and the rights of students with disabilities to obtain these technologies. The meaning of “Assistive Technologies” in this Act is tools, hardware, software or services for increasing the potential of disabled students. These technologies are not only designed for people with disabilities, but also adaptable or modifiable from mainstream methods (The Thai Ministerial Regulations for Provision of Assistive Technology, Media and Services in People with Disabilities, 2008). Thus, these technologies have the objectives of increasing, maintaining or developing abilities and potential for disabled persons, so that they are able to access information and activities in the same way as people in general. In Thailand, low technologies are gathered usually from local wisdom. They are not complex and mostly made from local products. On the other hand, high technologies involve more complex production and processes regarding their use, and their high cost means that they are used rarely by disabled people in the community. In this research, types of assistive technologies were categorized by kinds of users to form four groups of students with disabilities. Firstly, students with visual disabilities, such as low vision and blindness, were given assistive technologies that included environmental modification, text enlargement, contrast color of text and background, magnifiers, Closed- Circuit Television (CCTV), Brailler, Swing cell, Slate and stylus, Portable note takers, Optical Character Recognition (OCR), Screen reading program, white cane, infrared cane, etc. Secondly, students with hearing disabilities that related to communication and learning methods were given assistive technologies such as hearing aids, cochlear implant, FM system, loop system, Text Telephone or Telecommunication Device for the Deaf (TTY/TDD), Thai Telecommunication Relay Service (TTRS), Tone bar, Nasal indicator, Phonic mirror, Vocal 2 Programme, Speech viewer III and a Sign language interpreter. Thirdly, students with physical disabilities that related to daily living activities, maintaining posture, ambulation and mobility were given assistive technologies such as an electric tilt table, electric stand–in table and mobile stander, suction holder, food guards, walker, crutch, cane, wheelchair, hand-cranked bike, prosthesis, Augmentative and Alternative Communication (AAC), control interface, robotic aids, ergonomic keyboards, key guard, prism glasses, speech recognition, word prediction program, On-Screen keyboard, etc. Fourthly, students with intellectual disabilities that involved learning and daily living activities were given assistive technologies with shortcuts and easier steps. However, these technologies were not specially designed for this group of disabilities, but usually came from mainstream methods, such as the word prediction program, word completion and spell check program, Computer Assisted Instruction (CAI), digital pen and notebook, and educational board games (Deiner, 1993; Prongsantia, 2005; Saksiripol, 2005; Seephan, 2005; National Electronics and Computer Technology Center, 2006).

From the above information, some technologies can overlap with many types of disabilities, for example, the communication card, which can be applied to students with hearing, intellectual and physical disability as well as communication problems. Therefore, the Thai Ministerial Regulations for Provision of Assistive Technology, Media and Services for People with Disabilities regulates three types of assistive technologies, including “facilities”, “media”, and “services”, for disabled students. There are 25 assistive technologies in “facilities”, such as magnifiers, hearing aids, prostheses, cane, etc. There are 29 assistive technologies in “media”, such as sound books, big book, slate and stylus, sign language dictionary, communication board, fine motor skill development kit, etc. Finally, there are 20 assistive technologies in “services”, such as family counseling, nursing, and physical, occupational and speech therapy (The Thai Ministerial Regulations for Provision of Assistive Technology, Media and Services in People with Disabilities, 2008).

### 1.2 Specification of Problem

At present, the organizations that work with assistive technology in Thailand try continuously to develop new methods. Also, the educational organization that works for students with disabilities regulates the rights to access and obtain education and learning technologies. Bausch and Hasselbring (2004) reported that types of assistive technologies and related services were regulated many years ago in the Individuals with Disabilities Act Amendments P.L. 105–17. By following the regulations of the Act, more than 6 million students with disabilities could benefit via the Individualized Education Plan (IEP), which indicates types of disability and the rights to obtain assistive technologies.

However, although regulations and high technologies are developed continuously for students with disabilities, they do not necessarily bring them full benefits. Students could be helped to live with high technologies by adapting themselves because many assistive technology users would take more time to learn, train and practice their use. It would be more beneficial for disabled students if they used assistive technology until they have fully developed their skills. Besides the acceptance of using assistive technology, its provision is a source of concern and many problems such as the level of user knowledge, abilities and attitudes, lack of an evaluation process, ineffective planning, insufficient budget, complicated usage, limited time, and shortage of assistive technology professionals, especially in remote educational areas (Amatayakul, 1996; Lesar, 1998; Bausch & Hasselbring, 2004; Copley & Ziviani, 2004). Cooper et al. (2008) pointed out that not only modern or high technologies, but also problem trends in developing assistive technologies are related to new service delivery mechanisms, changes to public policy and coordination among consumers, policy-makers, manufacturers, researchers and service providers. These concerns highlight the ineffectiveness of these technologies.

Therapists play a role as a team for providing assistive technology, in terms of participation in needs evaluation, decision-making and implementing intervention. In this role, perspectives and opinions are given on the impact of assistive technologies on the recipients’ activities of daily living, education, play, leisure and social participation. The goal of this service is to ensure that disabled students and their family gain satisfaction from assistive technologies and continue to use them (Case-Smith, 2005).

In Thailand, many students with disabilities are diagnosed, and receive medical and educational rehabilitation services. They understand their rights to receive assistive technologies, especially for students in the school system, who have more opportunities to use them than those outside it. This is because the school team coordinates and works with related organizations in order to advocate these rights. However, assistive technologies cannot indicate that students will achieve full benefit from using them. As a result, the objectives of this research were to survey provision, frequency of use and needs for using assistive technologies, and analyze a model of providing them to disabled students in special education schools from the perspectives of key informants. The above can answer the following research questions: What kinds of assistive technologies were needed and provided for use by students with disabilities in special education schools and what was the organized system for providing them?

## 2. Method

This study design was quantitative and qualitative and conducted in Chiang Mai, Thailand. The instruments consisted of questionnaires and semi-structured interviews. Participants comprised students with disabilities, parents or caregivers of the students, teachers, school therapists and principals of special education schools.

One hundred and twenty students with disabilities (30 students each with visual, hearing, physical and intellectual disabilities), who studied in special education schools in Chiang Mai, Thailand, participated in this study. They comprised 73 males and 47 females, with 40 students each from kindergarten, primary school, and secondary or high school. Their ages ranged from 3 to 24 years. Most of the students were boarders and registered with a legal disability certificate, but surprisingly, 14 of them (11.70%) were not registered. Besides the students with disabilities, the participants in this study included four groups of key informants, i.e. 4 school principals, 4 teachers, 4 school therapists and 12 parents or caregivers of these students.

The instruments of this study were developed by researchers and processed for content validity by the suggestions from three related specialists. An Assistive Technology Checklist, or rating scale checklist, was developed by researchers and composed of three parts for provision, frequency and need to use assistive technology. The first part had a dichotomous scale (0 = no assistive technology provided in the school, 1 = had assistive technology provided in the school). The second part had a four-point scale (0 = not used, 1 = rarely used, 2 = sometimes used, 3 = frequently/always used). The third part had a dichotomous scale (0 = no need to use, 1 = need to use). In addition, the research instrument was semi-structured interviews with 1) problems and barriers in the provision and use of assistive technology and 2) an organized system to provide assistive technology to students. The interview questions were as follows:

1). In providing assistive technologies in your school, what will the process be in obtaining them for students with disabilities? Which organizations are related to, or work with you?
2). Do you have any problems or barriers during the above process? Do you have any problems or barriers in working with related organizations?
3). Do you have any problems or barriers in using assistive technologies, and if so what are they?
4). Do you have any opinions on the problems and barriers in obtaining assistive technologies in your schools?
5). Do you have any opinions on the problems and barriers in using assistive technologies in your schools?
6). What kinds of assistive technologies do you need for the students with disabilities in your school?
7). Which process do you consider effective for obtaining assistive technologies for the students with disabilities in special education schools?
8). Do you have any opinions on the provision of assistive technologies to students with disabilities in special education schools?
9). What are your expectations for the future regarding the provision of assistive technologies to students with disabilities in special education schools?

The participants for this study comprised 120 students with disabilities, 4 school principals, 4 school therapists, 4 teachers and 12 parents or caregivers. Permission to take part was granted by the principals of special education schools, i.e., schools for students with visual, hearing, physical and intellectual disabilities, in Chiang Mai, Thailand, and the participants signed a consent and assent form. Approval for this research was given by the Research Ethics Committee of the Faculty of Associated Medical Sciences, Chiang Mai University, Thailand. An Assistive Technology Checklist was gathered for 120 students with disabilities in order to survey the provision, frequency and need to use assistive technologies. An individual interview was administered directly to students with good communication skills in the Thai language, but caregivers were given the interview instead of students who were too young or had communication problems. The four groups of key informants gave data via the semi-structured interviews, which included the problems and barriers in the provision, use, and point of an organized system in providing assistive technology to students.

Quantitative data were analyzed by descriptive statistics for analyzing the use, problems and barriers in using assistive technologies for students with disabilities in special education schools. Qualitative data were analyzed by content analysis to find a model of providing assistive technologies to such students.

The process of the research method is as follows:

**Figure 1:**
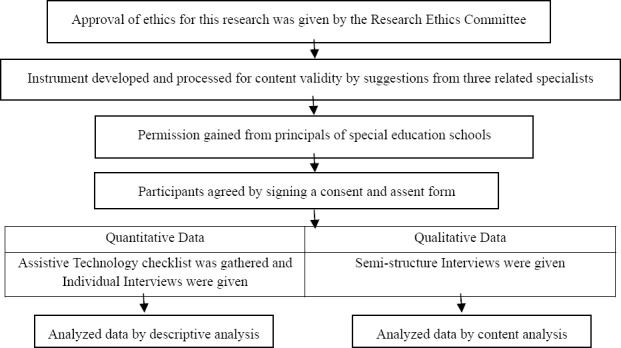
The process of the research method

## 3. Results

The results indicated that all types of assistive technologies in this study were provided to students with disabilities in special education schools. The types of assistive technology provided were “service”, “media” and “facility”, consecutively. Mostly students with physical disabilities were provided, but those with hearing disabilities were provided the least. In addition, the results showed that the most provided “facility”, “media”, and “service” was screen reading software, computer assisted instruction (CAI) software and electronic educational media, and nursing, respectively.

The results indicated that students with visual and physical disabilities used assistive technologies the most and least, respectively. When considering the types of assistive technologies used by the students, it was found that those with visual and hearing disabilities used “service” more than the other types, but those with physical and intellectual disabilities used “media” more than the others. However, the level of use in students with physical, visual and hearing disabilities was at the low level (rarely used), while the level of use in students with intellectual disabilities was at the moderate level (sometimes used). Furthermore, the results revealed that all groups of students used “facility” at the low level. The students with physical, visual and hearing disabilities used “media” at the low level, while those with intellectual disabilities used it at the high level. Regarding “service”, the students with hearing disabilities used it at the low level, while those with visual disabilities used it between the low and moderate level, and those with physical and intellectual disabilities used it at the moderate level. When each type of assistive technology was considered, screen-reading software, electronic educational media and fine motor skill program, and nursing were the “facility”, “media”, and “service” used by most students, respectively (Table 1).

**Table 1:** Level of use by type of assistive technology

Students	% of use FAC			% of use MED			% of use SEV			% of use total		
			
	RL	ST	FQ	RL	ST	FQ	RL	ST	FQ	RL	ST	FQ
PD	53.8	23.1	23.1	81.5	18.5	0.0	35.3	47.1	17.6	61.4	28.1	10.5
VD	85.0	0.0	15.0	85.2	7.4	7.4	40.0	40.0	20.0	71.6	14.9	13.4
ID	57.1	21.4	21.4	27.6	20.7	51.7	26.3	68.4	5.3	33.9	35.5	30.7
HD	71.4	14.3	14.3	73.1	19.2	7.7	70.0	15.0	15.0	71.7	16.7	11.7
All	72.0	8.0	20.0	86.2	10.3	3.4	40.0	35.0	25.0	68.9	16.2	14.9

*Note.* PD = students with physical disabilities; VD = students with visual disabilities; ID = students with intellectual disabilities; HD = students with hearing disabilities; FAC = Facility; MED = Media; SEV = Service; RL = Rarely used; ST = Sometimes used; FQ = Frequently used.

The results showed that students with visual disabilities needed assistive technology the most, but those with hearing and physical disabilities needed it the least. Indeed, a laptop computer, computer assisted instruction (CAI) program, and nursing was the “facility”, “media”, and “service” most needed by the students, respectively (Table 2). Table 2 shows an overview of the provision, use and needs of assistive technology for students with disabilities in special education schools.

**Table 2:** Provision, use and needs of assistive technology for students with disabilities in special education schools

Students	% of Provision				% of Use				% of Needs			
		
	FAC	MED	SEV	TOTAL	FAC	MED	SEV	TOTAL	FAC	MED	SEV	TOTAL
PD	68.0	96.6	100.0	87.8	52.0	93.1	85.0	77.0	64.0	93.1	100.0	85.1
VD	72.0	89.7	95.0	85.1	80.0	93.1	100.0	90.5	96.0	100.0	95.0	97.3
ID	56.0	89.7	100.0	81.1	56.0	100.0	95.0	83.8	76.0	93.1	100.0	89.2
HD	48.0	93.1	100.0	79.7	56.0	89.7	100.0	81.1	56.0	100.0	100.0	85.1
All	96.0	100.0	100.0	98.7	100.0	100.0	100.0	100.0	100.0	100.0	100.0	100.0

*Note.* PD = students with physical disabilities; VD = students with visual disabilities; ID = students with intellectual disabilities; HD = students with hearing disabilities; FAC = Facility; MED = Media; SEV = Service.

The results of qualitative data came from the interviews with four groups of key informants, such as parents or caregivers, teachers, school principals and school therapists. The information given could be separated into three points: sources of assistive technology for students with disabilities in special education schools; the problems and barriers in the provision and use of assistive technology in special education schools; and the perspective on a model of providing assistive technology to the students in question (Table 3). The key informants pointed out three sources of assistive technology, i.e. government and private organizations and schools. The schools were provided with a budget from the government to build a new building, but it did not include provision of assistive technologies. This was due to the regulation that schools provided with assistive technologies had to be mainstream and not special education schools. Therefore, special education schools needed to produce a project submitted directly to the government in order to receive assistive technologies for their students, for example, the project to obtain special shoes or leg braces from Sirindhorn National Rehabilitation Centre for students with physical disabilities. Despite that project being passed and the schools concerned receiving assistive technologies, they were insufficient for all of the students. In addition, the collaboration of government organizations came from educational institutes, such as Chiang Mai University and Rajamangala University of Technology Lanna, which had senior projects by students on innovative technologies for students with disabilities. After completion of the projects, their technologies were given to the students. After that, the pattern of collaboration came from the charities of private national and international organizations, for example, the Anusarnsunthorn Foundation, Northern Association of the Blind, international collaboration projects from Japan and Singapore, Thai donations made by parent groups of children with disabilities, which helped families of children with disabilities, donations from mature students, and company projects such as those from IBM, True Corporation, etc. Finally, school budgets did not only come from the government, but also community donations that were used to provide assistive technologies through purchasing local mainstream technologies, and using materials with which to make low-tech assistive technologies themselves. The schools also hired tutoring teachers, assistive technology technicians and other related professionals.

**Table 3:** Perspectives of the key informants on provision, use, and needs of assistive technologies

Key informants	Perspectives		

	Sources of assistive technology (AT)	Problems and barriers	Model for providing assistive technology (AT)
Parents or caregivers	• Government • Private organizations • Schools	• Lack of knowledge and understanding about AT • Information about a repair and maintenance center • More skills needed for using and maintaining AT	• Collaboration • Learning systems and production manual for users • Developing an AT center
Teachers	• Government • Private organizations • Schools	• Many responsibilities in schools • Lack of knowledge and understanding about AT • Information about a repair and maintenance center • More skills needed for using and Maintaining AT	• Collaboration • Learning systems and production manual for users • Developing an AT center
School principals	• Government • Private organizations • Schools	• Educational policy • Insufficient technologies for individual students	• Collaboration • Independent management of the schools • Developing an AT center
School therapists	• Government • Private organizations • Schools	• Many responsibilities in the schools	• Collaboration • Holistic perspective • Learning systems and production manual for users • Developing an AT center

An important problem in the provision and use of assistive technology was the criteria of educational policy, which indicated that students with disabilities, who studied in regular or mainstream schools, would have the rights to obtain assistive technologies as indicated in their IEP. On the other hand, this policy did not cover students who were studying in special education schools. Therefore, these schools would have the responsibility of managing their own budgets in order to provide assistive technologies for their students, particularly when providing “service” because the schools usually obtain “facilities” and “media” from charity organizations. They rarely obtain funds for providing “service”, for example; hiring school therapists, nurses, assistant teachers, etc. This reflected on the lack of ability for schools to provide professionals with an adequate salary that would encourage incentive and motivation. Furthermore, school professionals had too many responsibilities, which made their work ineffective. In addition, the lack of professionals caused inadequate knowledge and understanding of assistive technology and its maintenance in users that included students with disabilities, parents or caregivers and teachers. Many teachers reported that they did not know about some assistive technologies or how to use them. Additionally, it was not known which assistive technology matched or suited which group of students with disabilities. Therefore, when assistive technology was written in the IEP, it was not determined in the plan. Parents or caregivers and teachers reported that they had no knowledge about the maintenance of assistive technology at home and in schools. Also, if the assistive technology developed problems or failed to work, they did not know where to go for repairs or assistance because most assistive technologies were imported and had short-term guarantees. When having a problem, it took more time to order parts or send away for repairs. Indeed, the cost of repairing and sending abroad was high. In fact, limitations in the number of assistive technologies obtained by schools prevented provision to individual students. This caused a lack of opportunities for students to use assistive technology continuously, and created barriers in developing skills for using it. Therefore, this situation could be a reason why many students with disabilities felt that assistive technologies were not useful or too complex to use, which ultimately brought about their rejection.

From the perspective of students with disabilities, an effective system for the model of providing assistive technology in special education schools was outlined by key informants, who pointed out five factors as follows:

1). Collaboration

Collaboration should involve all related organizations, including government and private organizations, and communities. However, the government organizations related to special education should be major ones that give support in providing a system for assistive technologies. In addition, organized collaboration should encourage students with disabilities and their families to participate in the process of decision-making, training and using assistive technologies.

2). Holistic perspective

In providing assistive technologies, the needs of students with disabilities should be considered in all areas of human occupations, such as activities of daily living, education, leisure and social participation.

3). Independent management of schools

Schools should adopt various methods of management for providing assistive technologies to students without delay, due to limited budgets and lack of assistive technology specialists. An example of management is encouragement for teachers in schools to participate in technician courses that can enable them to solve simple problems and give students, their parents and teachers information about the maintenance of assistive technologies. Another example is adapting old computers for use as teaching media in the school computer room, instead of acquiring a new computer from government or other organizations.

4). Learning systems and production manual for users

After the students with disabilities are provided with assistive technologies, they should participate in a learning course in order to receive knowledge and information about using and maintaining assistive technologies

5). Development of an Assistive Technology Center

The Assistive Technology Center has been developed as an organization that plays the roles of systematically surveying and evaluating the use of assistive technologies. The role of surveying is useful for the policy-makers in knowing the needs and sufficiency of assistive technologies for the students. In addition, the role of evaluation is for gathering information and highlighting problems after receiving and using assistive technologies, both by individuals and as a group. Furthermore, this center should have roles of advocacy in order to produce, provide, train in, repair and maintain assistive technologies, and possess a course for assistive technology specialists because this technology has specific and complex characteristics that need professionals who are highly-skilled to produce and repair it. However, there are no assistive technology specialists in Thailand that can indicate the characteristics of effective assistive technologies, and follow up their use after they have been provided. Rectifying this factor might help to solve the problems of inadequate assistive technology sources.

## 4. Discussion

The results found that although special education schools were not regulated to receive assistive technologies, the students with disabilities in all four schools received almost all of them. This is because these schools had been set up for a long time in Thailand, and their administrators understood the process for requesting assistive technologies from the government and network organizations, which was by producing projects. This result related to a study by Carlson and Ehrlich (2006), who reported that there was more than one source of payment for providing assistive technology to persons with disabilities. In this study, mostly students with physical disabilities were provided with assistive technologies because of their obvious handicap. On the other hand, the students with hearing disabilities were provided with assistive technologies the least, because this type of handicap is not obvious until they try to communicate. Most of the students with hearing disabilities in special education schools were deaf, and although regulated for assistive technologies, they only received analog hearing aids, which were inappropriate for use in the classroom. In fact, deaf students needed sign language interpreters as a type of “service” instead of hearing aids, and so many students with analog hearing aids did not use them.

Students with visual disabilities used assistive technology the most, but those with physical disabilities used it the least. This contradicted the results from providing assistive technology, which indicated that mostly students with physical disabilities were provided with assistive technologies. The reason for this could be explained by most of the assistive technologies being mobility ones for students with physical disabilities, which agreed with results from Carlson and Ehrlich (2006), who found that the largest assistive technology component was made up of mobility devices. However, when these devices did not work, the schools had no budgets for repairs, and eventually the technologies became useless. Besides mobility technologies, students with physical disabilities were provided with learning technologies, especially computers, but these students had physical limitations when using them without adaptation or modification. As a result, the students with physical disabilities faced barriers in accessing assistive technologies and their use decreased more in them than in students with other disabilities.

When considering frequency of use, it was found that all types of assistive technologies were used at the low level, and had the trend of change or complete rejection if students perceived that assistive technologies were too difficult to use. This point of discussion related to the study of Lesar (1998), who reported that most students with disabilities were concerned about knowledge and the ability to use their assistive technologies. Also, they gave importance to training and practicing in the use of assistive technologies before and during the school term.

## 5. Conclusion

In this study, the model of providing assistive technology to students with disabilities in special education schools comprised 5 components: Collaboration; Holistic perspective; Independent management of the schools; Learning systems and production manual for users; and Development of an assistive technology center, which was driven by 3 major sources such as Government and Private organizations, and Schools. This model is related to the study of Copley and Ziviani (2004), who suggested a Team Model for decreasing the barriers in using assistive technologies in children with disabilities. In addition, Phetmoo (1995) suggested that the role of therapists or specialists in areas of needs evaluation, counseling and training, utilize assistive technologies, which is related to this study.

However, this study had limitations because it was carried out ‘in Chiang Mai province only’, which might not be a general indicator for other areas in Thailand, as they have different support, barrier and cultural factors. In future study, researchers may examine each group of students with disabilities in a wider area and in greater detail.

## References

[ref1] Amatayakul P (1996). Special education and modification of technology to children with disabilities.

[ref2] Bausch M. E, Hasselbring T. S (2004). Assistive Technology: Are the Necessary Skills and Knowledge Being Developed at the Preservice and Inservice Levels?. J Teach Edu.

[ref3] Carlson D, Ehrlich N (2006). Sources of Payment for Assistive technology: Finding From a National Survey of Persons with Disabilities. Assist Techno.

[ref4] Case-Smith J (2005). Occupational Therapy for Children.

[ref5] Chester M. D (2012). Access to Learning: Assistive Technology and Accessible Instructional Materials.

[ref6] Cooper A. R, Cooper R, Boninger M. L (2008). Trends and Issues in Wheelchair Technologies. Assist Techno.

[ref7] Copley J, Ziviani J (2004). Barriers to the use of assistive technology for children with multiple disabilities. Occup Ther Int.

[ref8] Deiner P. L (1993). Resource for teaching children with diverse abilities.

[ref9] Grand Rapids Autism Program (2003). Parent's Role in Transition Planning.

[ref10] Lesar S (1998). Use of Assistive Technology With Young Children With Disabilities: Current Status and Training Needs. J Early Interv.

[ref11] National Electronics and Computer Technology Center (2006). Survey Report of Needs of Information Technology for People with Disabilities in Thailand.

[ref12] Phetmoo K (1995). A survey of needs and use of activity of daily living devices in elderly people.

[ref13] Prongsantia S, Niyomdham S (2005). Technology for Children with Visual Disability. Technology for Children with Disability 1.

[ref14] Saksiripol D, Niyomdham S (2005). Technology for Children with Hearing Disability. Technology for Children with Disability 1.

[ref15] Seephan S, Niyomdham S (2005). Technology for Children with Physical Disability. Technology for Children with Disability 1.

[ref16] The Thai Ministerial Regulations for Provision of Assistive Technology, Media, and Services in People with Disabilities (2008). The Thai Ministerial Regulations for Provision of Assistive Technology, Media, and Services in People with Disabilities B.E. 2550.

